# Inverted ductal papilloma of the oral cavity secondary to lower lip trauma. A case report and literature review

**DOI:** 10.4317/jced.51055

**Published:** 2013-04-01

**Authors:** Sergi Sala-Pérez, Antoni España-Tost, August Vidal-Bel, Cosme Gay-Escoda

**Affiliations:** 1 DDS. Resident of the master of oral surgery and implantology. Barcelona University Dental School; 2 MD, DDS, PhD. Associate professor of oral and maxillofacial surgery. Professor of the master of oral surgery and implantology. Barcelona University Dental School. Investigator of the IDIBELL Institute; 3 MD. Pathologist of Bellvitge University Hospital. Associate professor of the department of pathology and experimental therapeutics of the university of Barcelona. Investigator of the IDIBELL Institute; 4 MD, DDS, PhD. Chairman of oral and maxillofacial surgery. Director of the master of oral surgery and implantology. University of Barcelona Dental School. Coordinating investigator of the IDIBELL Institute. Head of the service of maxillofacial surgery, Teknon Medical Center. Barcelona, Spain

## Abstract

Inverted ductal papilloma of the oral cavity is an infrequent benign neoplasm of papillary appearance that originates in the secretory duct of a salivary gland. The etiology is unknown, though some authors have related it to human papillomavirus (HPV) infection. We present the case of a 40-year-old woman with a tumor of the lower lip mucosa. Histopathological study of the lesion diagnosed inverted ductal papilloma of the oral cavity. Human papillomavirus DNA detection and typing based on tumor lesion DNA amplification and posterior hybridization, revealed no presence of viral DNA. The antecedents of trauma reported by the patient could have played an important role in the development of this tumor.

** Key words:**Inverted ductal papilloma, intraductal papilloma, oral papilloma, papillary epidermoid adenoma.

## Introduction

Oral papilloma is a benign tumor located in the oral mucosa. It normally shows exophytic growth with a poly-poid or verrucous appearance. However, there have been reports of cases characterized by endophytic or inverted growth (1), most often located in the nasal and paranasal sinus mucosa, in the bladder, and in the lacrimal gland (2). Some papillary lesions of the secretory duct of a salivary gland, such as oncocytoma, papillary cystoadenoma, Warthin’s tumor, intraductal papilloma (IP), inverted ductal papilloma and sialoadenoma papilliferum can clinically manifest as tumors of the oral cavity (3). Inverted ductal papilloma of the oral cavity (IDPOC) is an infrequent benign neoplasm of papillary appearance that originates in the secretory duct of a salivary gland. It forms part of a group of ductal papillary neoplasms, together with intraductal papilloma and sialoadenoma papilliferum. The size of the lesion varies from 0.5-1.5 cm in diameter, and the most common location is in a minor salivary gland of the lip and lower cheek mucosa (2,3). The etiology is unknown, though some authors have related it to human papillomavirus (HPV) infection, due to the detection of HPV sequences (subtypes 6/11) within the lesion (4,5). Certain immunohistochemical and ultrastructural studies have suggested that IDPOC may originate in the transition zone between the distal extremity of the secretory duct of a salivary gland and the surface epithelium of the oral cavity (6). Surgical removal is the treatment of choice for IDPOC. No relapses or malignant transformations have been reported to date (3,7).

## Case Report

**Figure 1:**
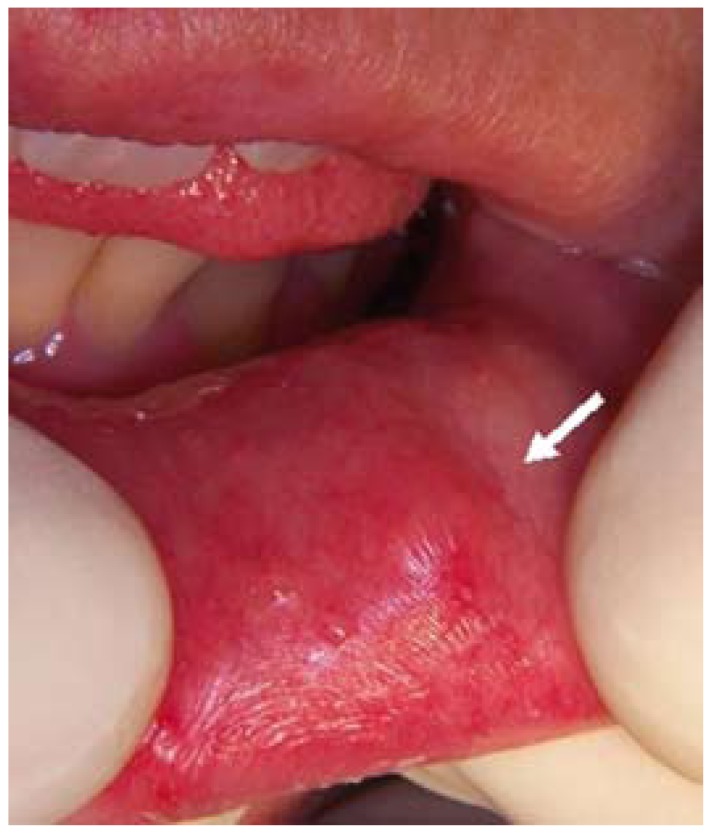
Zone affected without signs of inflammation or suppuration.

A 40-year-old caucasian woman was reported to our service of Oral Surgery for evaluation and treatment of a tumor located in the lower left lip mucosa. She had the habit of nibbling her lower lip, and had suffered trauma in this same region, upon impacting with the head of her young child. After three months the patient noticed slight changes in the size of the lesion. She appeared to be in good health, and no maxillary asymmetries or tumors, or neck adenopathies were detected. At clinical exploration, the lesion showed a nodular aspect, of soft consistency, with no adherence to deep layers, and no pain in response to palpation (Fig. 1). There were no signs of inflammation or suppuration in the affected zone. The rest of the orofacial structures (teeth, upper lip, mucosal membranes, gums, tongue, floor of the mouth and mandible) were normal. The panoramic X-ray study likewise showed no alterations.

- Differential diagnosis

Based on the location, the history of trauma and the clinical characteristics of the lesion, a lower lip mucocele was initially diagnosed. However, the differential diagnosis also included other conditions such as pleomorphic adenoma, traumatic fibroma and lipoma.

- Diagnosis and treatment

Complete removal of the lesion was carried out with the CO2 laser under local anesthesia using 4% articaine with 1:100.000 adrenalin (Laboratorios Inibsa, Barcelona, Spain). The specimen sent to the pathology laboratory was of a pinkish color, rounded and measured 1 x 0.5 cm in size. The following postoperative medication was prescribed: ibuprofen 600 mg in tablets, one every 8 hours for 5 days (Ibuprofeno 600 mg, Zambom, Barcelona, Spain) and chlorhexidine gel, three applications a day for 10 days (Clorhexidina Gel Bioadhesivo 50 ml, Lacer, Barcelona, Spain). The postoperative course was free of complications, and the patient is presently subjected to periodic controls to detect possible relapse.

- Histological study

**Figure 2:**
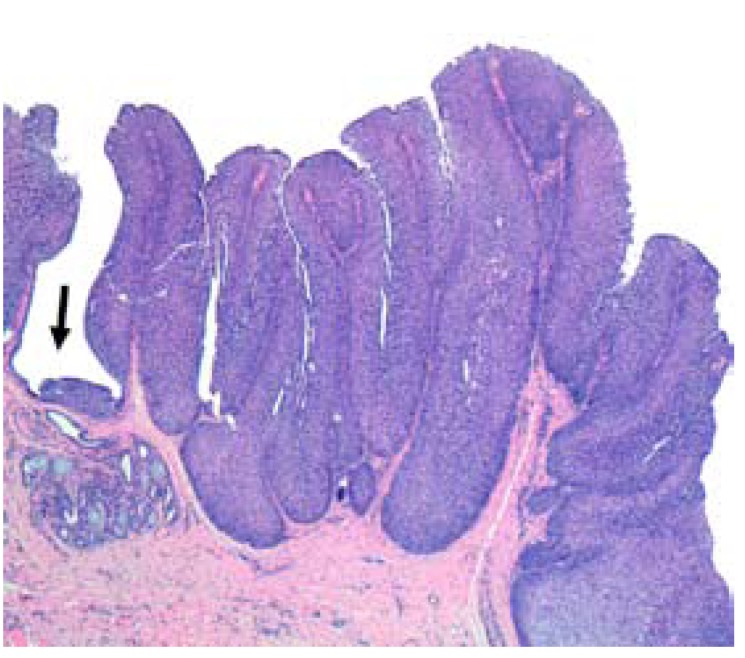
lesion located between the secretory duct of a minor salivary gland and the surface epithelium of the oral cavity.

**Figure 3:**
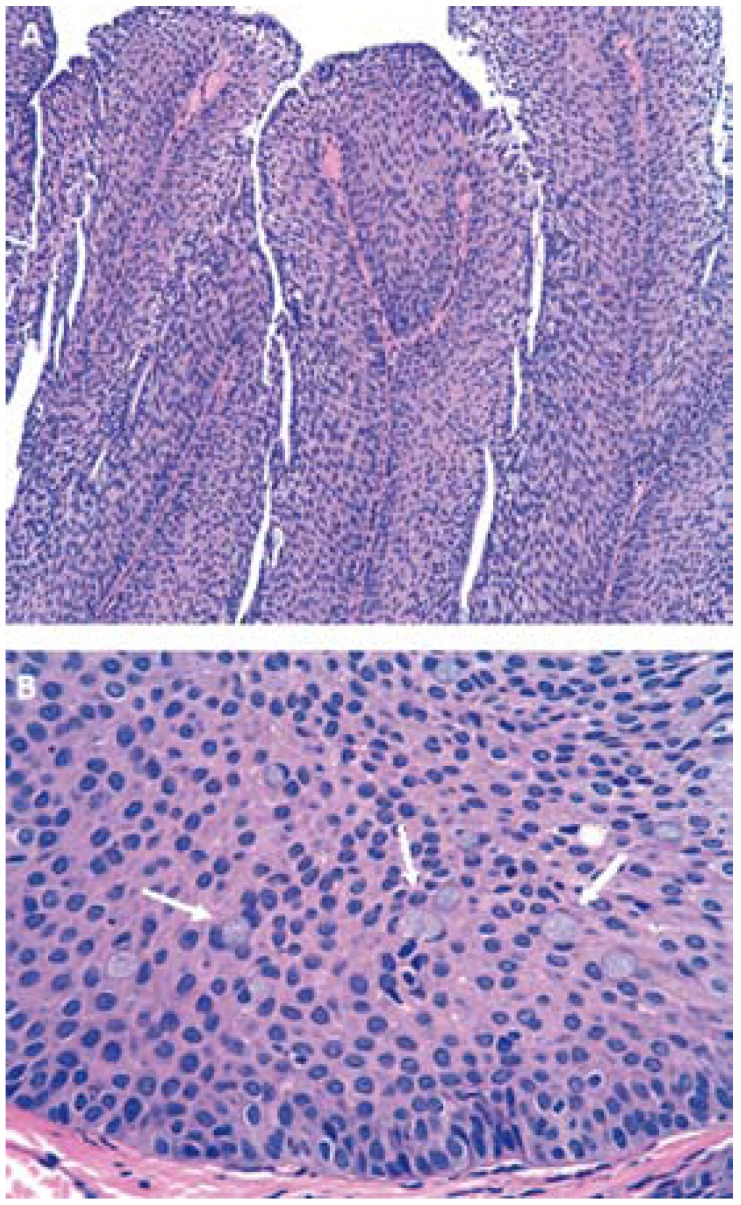
A. Tumor composed of basaloid squamous cells without cytological atypia; B. Included isolated mucosecretory cells.

The surgical specimen was stained with hematoxylin-eosin (HE) and examined under an Olympus CX31RBSF light microscope (Olympus Corporation, Tokyo, Japan). The histological study revealed an endophytic lesion with extensive, rounded and regular margins, exhibiting an expansive growth pattern. The lesion was located in the transition zone between the secretory duct of a minor salivary gland and the surface epithelium of the oral cavity (Fig. 2). The tumor was composed of basaloid squamous cells without cytological atypia and included isolated mucosecretory cells (Fig. 3). Based on these findings, inverted ductal papilloma of the oral cavity was diagnosed.

- Detection of HPV

DNA extraction was carried out from the sample fixed in formalin solution and embedded in paraffin, based on the conventional technique. HPV detection was carried out with the HPV LA genotyping test (Roche Molecular Systems Inc., Los Angeles, CA, USA). This process involves amplification of the viral DNA sequence via polymerase chain reaction (PCR) and posterior hybridization using probes corresponding to a total of 37 HPV subtypes – 18 of which are considered to be of high risk (16, 18, 26, 31, 33, 35, 39, 45, 51, 52, 53, 56, 58, 59, 66, 68, 69 and 82), and 19 of low risk (6, 11, 40, 42, 54, 55 , 61, 62, 64, 67, 70, 71, 72, 73, 81, 83, 84, IS39 and CP6108). The procedure for typing HPV DNA was carried out based on the same methodology as described above, and using the primers PGMY 09/11 for viral DNA amplification, and the primers PCO3, PCO4 and PCO5 for amplifying the β-globin gene, which was used as internal control. The biotinylated amplicons were denaturalized with 0.4 N NaOH and hybridized in an immobile matrix with probes corresponding to 37 different HPV subtypes, according to the protocol recommended by the manufacturer (Roche Molecular Systems Inc., Los Angeles, CA, USA). Hybridization positivity was detected by streptavidin – horseradish peroxidase precipitation onto the probe membrane. The sample DNA was correctly amplified, though no HPV DNA was detected.

## Discussion

Inverted ductal papilloma of the oral cavity (IDPOC) was first described in 1982 by White et al. (2) as a benign tumor located in the secretory duct of a salivary gland, though the year before Basatkis et al. (8) had published three cases of an identical lesion which they referred to as papillary epidermoid adenoma. Due to its histological similarity to intraductal papilloma (IP) of the sinusal, nasal, bladder and oral mucosa, White et al. (2) coined the term “inverted ductal papilloma of the oral cavity” in reference to this lesion. IDPOC is an infrequent tumor of uncertain incidence (3). Regezi et al. (9) detected four of these lesions in a series of 238 minor salivary gland tumors. This has been the first and the only case of IDPOC diagnosed out of a total of 90 minor salivary gland biopsies performed in our service of Oral Surgery in the period 2003-2009. Since 2001, then there have been reported 10 new cases of IDPOCs, thus representing a total of 44, including the present case ( Table 1).

**Table 1:**
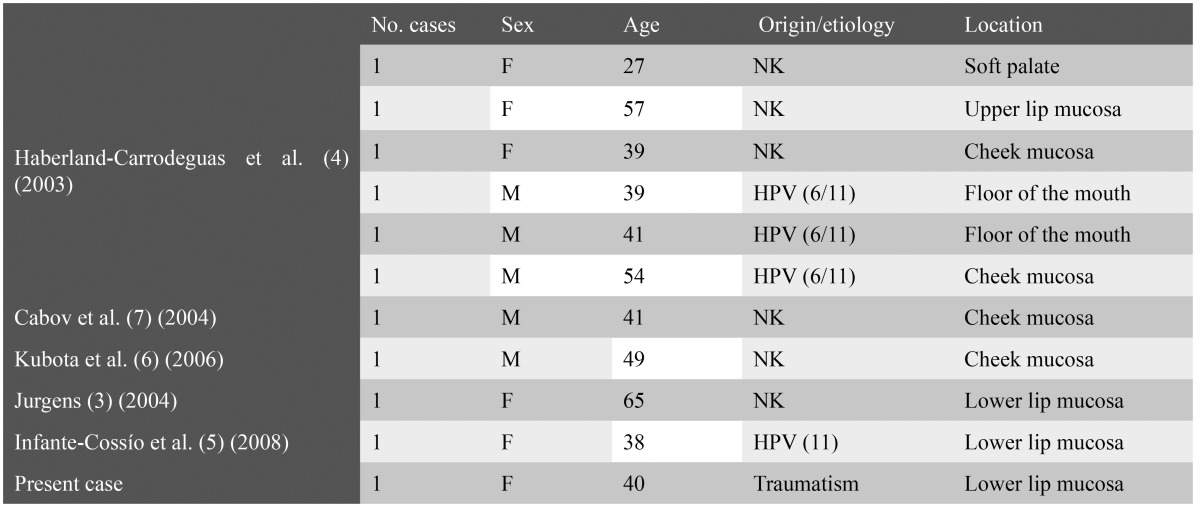
New cases of inverted ductal papilloma of the oral cavity published since 2001 and up to the present case. F = female; M = male; HPV = human papillomavirus; NK = not known.

The patient age at appearance of the lesion is usually between the fourth and fifth decade of life and no particular gender predilection has been observed (1,5). In our review of the literature, the mean age was found to be 44 years, with a range of 27-65 years. Clinically, IDPOC manifests as a submucosal tumor of nodular appearance and with a smooth or verrucous overlying surface, where dilatation of the salivary gland secretory duct outlet is generally noted (3). The lesion normally communicates with the mucosal surface (6), though a separating fibrous connective tissue band may be present. In our case the patient reported changes in the size of the lesion due to the internal accumulation of mucoid material. The oral structures most commonly affected by these lesions are the minor salivary glands – particularly those of the lower lip mucosa, cheek mucosa and floor of the mouth(3,5). The etiology is of IDPOC unknown, though some authors have related it to human papillomavirus (HPV) infection. Haberland-Carrodeguas et al. (4) were able to isolate HPV subtypes 6/11, while Infante-Cossío et al. (5) detected HPV subtype 11 in an HIV-infected female. In our case HPV DNA was not detected in the lesion, and no cytopathic changes suggestive of viral infection of the epithelial cells were observed. The antecedents of recurrent trauma reported by the patient in the affected zone possibly could have played an important role in the development of this tumor. In the latest cases reported in the literature, the mean evolution of the lesion has been 3-6 years, with a location in areas habitually exposed to trauma, such as the lip mucosa (as in our patient), the cheek mucosa and floor of the mouth. Macroscopically, IDPOC is an organized, non-encapsulated lesion presenting papillary crests that can form multicystic internal spaces. Growth is towards the lumen, and the tumor can even spread in an organized manner beneath the underlying connective tissue. Based on the location of the lesion, its growth pattern and papillary appearance, the histological differential diagnosis must be established with sialoadenoma papilliferum and intraductal papilloma (IP). However, from the histopathological perspective, the presence of mucosecretory cells among the basaloid squamous cells requires a differential diagnosis with mucoepidermoid carcinoma (6,7). Sialoadenoma papilliferum is distinguished from IDPOC by its exophytic growth. This papillary lesion presents inflammatory connective tissue projections with acanthosis and parakeratosis at the surface of the epithelium. The gland stroma component also forms papillary prolongations with columnar or cuboid cells and mucosal cells. On the other hand, intraductal papilloma (IP) is an endophytic tumor that grows inwards within the duct, though in contrast to IDPOC, it forms a unicystic cavity. Mucoepidermoid carcinoma in turn is characterized by the presence of basal squamous cells and mucosal cells. The low grade lesions may present scant cytological atypia and pose differential diagnostic problems with IDPOC – particularly in the case of incisional biopsies, where the characteristic structural pattern of the disease cannot be appreciated. A complete lesion sample is thus required in order to avoid diagnostic error (8). The treatment of choice is complete surgical removal, followed by histological analysis. In our case we used the CO2 laser for resection due to report fewer complications and relapses than the cold scalpel. The postoperative course in our patient was free of complications, and she is presently subjected to periodic controls to detect possible relapse. No relapses or malignization of IDPOC have been documented though a squamous cell carcinoma in an IP of the cheek mucosa has been identified (1). In most cases these complications are a consequence of an incomplete resection of the lesion.
